# A walk in the country: storying rural journeys of dementia care

**DOI:** 10.1093/geroni/igab046.3554

**Published:** 2021-12-17

**Authors:** Jami Horne, Katie Aubrecht

**Affiliations:** St. Francis Xavier University, St. Francis Xavier University, Nova Scotia, Canada

## Abstract

Dementia and dementia caregiving are increasingly recognized as significant public health issues. Dementia may be more prevalent in rural communities; in part due to higher rates of population aging. In Canada the Nova Scotia provincial dementia strategy Towards Understanding (2015) emphasizes the need to address the unique realities of rural dementia care as a priority issue; however, research remains limited on this demographic in the province, and Atlantic Canada more broadly. This presentation shares findings from the Royal Bank of Canada Foundation funded study, Rural Dementia Caregiving: A Community Life Story, conducted in 2021 to address this critical knowledge gap. The qualitative research design involved a narrative review, archival research and narrative analysis of interviews that yielded rich stories of family/friend dementia caregiving in rural Nova Scotia. Stories illustrate how history, culture and identity inform dementia caregiver realities, experiences and self-perceptions. Study results also suggest that rural dementia caregiving is characterized by factors that include strong community networks and deep-rooted connections to land, culture, and heritage, which can be experienced as supportive as well as constraining. The conditions of life in rural communities, including restricted access to internet, transportation, essential services and paid care providers, pose challenges to dementia caregiving. They also provide opportunities in which networks and connections become more visible and may even be strengthened. Findings demonstrate the lived realities of rural dementia caregivers and the people they care for are unique. Addressing their needs require a distinct approach that acknowledges and can appropriately respond to these differences.

